# Optically Graded Ultra Dark Absorber for Visible and Near-infrared Wavelength Range

**DOI:** 10.1038/s41598-018-30844-5

**Published:** 2018-08-17

**Authors:** Prabhat K. Agnihotri, Viney Ghai, Harpreet Singh

**Affiliations:** Department of Mechanical Engineering, Indian Institute of Technology Ropar, Nangal Road, Rupnagar, 140001 Punjab India

## Abstract

Near perfect absorbers find application in many areas including solar cells, energy harvesting and antireflection coatings for space applications. Here we report the use of optical gradation concept to fabricate a near perfect absorber on etched Si wafer. As a proof of concept, 99.4% absorption is achieved in the broad range of 300 nm to 2000 nm. Moreover, absorption capacity of optically graded surface remains higher than 99% up to beam incident angle of 50°. While carbon nanotubes (index ~1.1) are used as top layer, subsequent layers with increasing optical index across the thickness are chosen so as to satisfy zero reflection condition on multilayered assembly. Inward bending of incident beam and total internal reflection of reflected beam caused due to optical index gradient contributes to absorb the incident beam more efficiently. In addition, multiple scattering of incident beam due to the presence of multiscale feature size in graded assembly helps to absorb shorter as well as longer wavelengths of incident light. The graded assembly shows contact angle of 160° with roll-off angle equal to 5° implying that the graded absorber is not only super black but also superhydrophobic and self-cleaning in nature. The combination of properties shown by the super absorber makes it very attractive, especially for next generation solar cells to harness energy in the wavelength range of 1000 nm to 2000 nm.

## Introduction

Super black absorbers are attractive in many technologically important areas such as photovoltaics, stealth technology and antireflection coatings for space and defence applications^1–4^. A super absorber should ideally have extremely low reflectance (*R* < 0.2%) and very high absorption (A > 99%) over broadband frequency range and angle of incident beam^5^.Various processes as well as materials have been developed to bring down the refractive index of objects to unity and thus increase their absorption capacity^6–9^. It is reported that introduction of hierarchal micro-nano structures leads to a near perfect absorber^10–12^. The absorbing frequency range of these hierarchal structures depend on the number of factors including refraction index, texture shape, size and their arrangement in the material microstructure^13^. Synthesis of extremely dark material from low density carbon nanotubes arrays^14^ (*n* = 1.01–1.1) has shown an excellent combination of low reflectance and high absorbance. Use of nanostructures and metamaterials has also been reported to make better absorbers^15,16^. However, application of metamaterials is limited due to the challenges associated with their fabrication and processing cost. Recently, efforts have been made to harness the energy in 1000 nm to 1400 nm wavelength range in next generation solar cells^17^. In spite of these advancements, most of the reported absorbers are effective in a particular wavelength and incident beam angles^16,18,19^. Consequently, there remains a need to develop a near perfect absorber which can efficiently work in broader wavelength range of 300 nm–2000 nm and at varying incident beam angles. We report herein the design and fabrication of an optically graded superhydrophobic and self-cleaning surface with near perfect absorption (99.4%) in the broad wavelength of 300 nm to 2000 nm with incident beam angle in the range of 0–60°. Fabrication of such an absorber has not been so far realized to the best of our knowledge.

**Figure 1:**
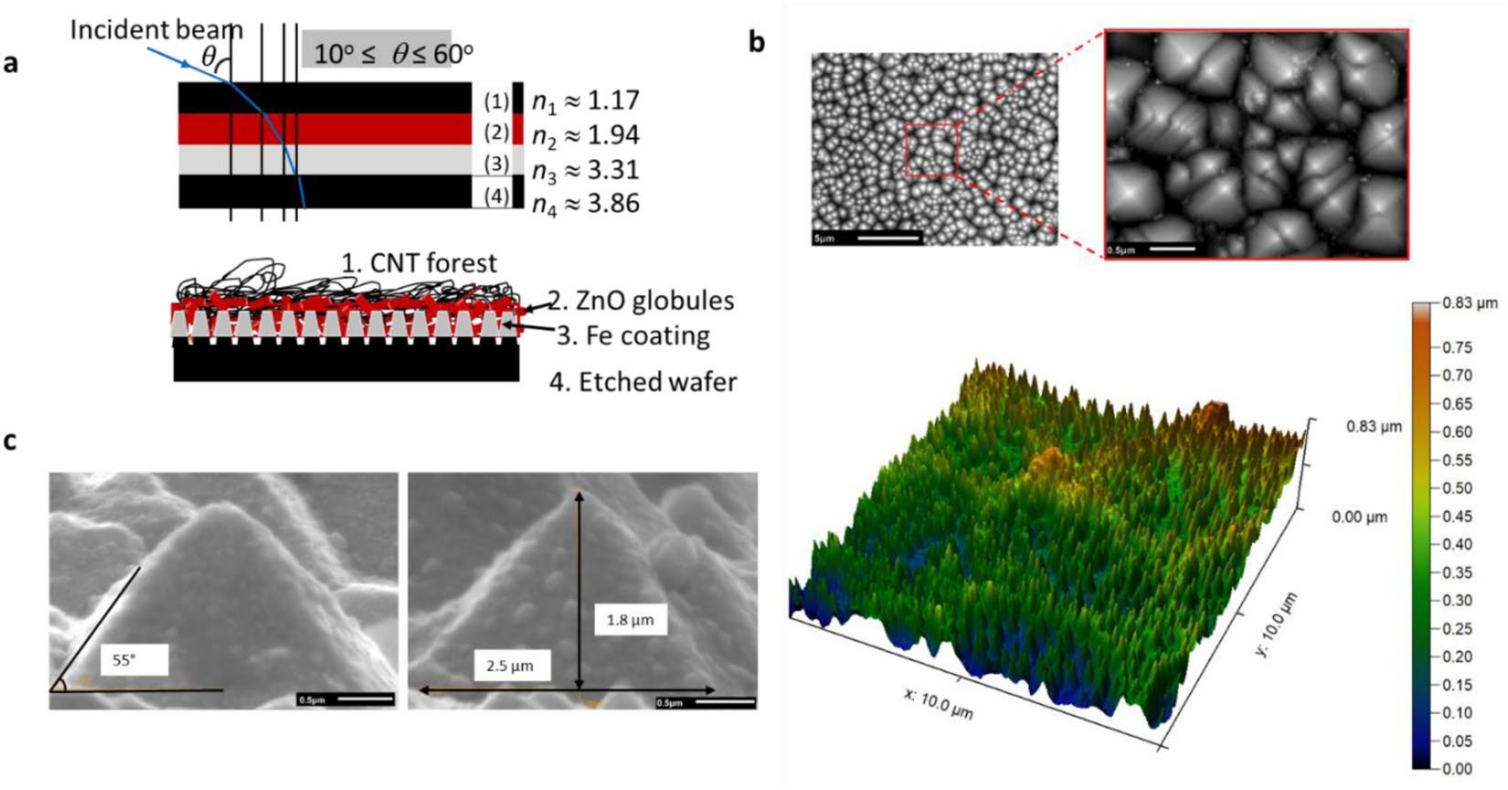
Schematic of optically graded assembly with SEM and AFM of etched P-type silicon wafer. (**a**) Shows the design approach used to create optically graded surface with multiscale feature size and increasing optical index in the direction of incident beam. CNT forest as top most layer is used to ensure impedance matching and Si wafer is used as substrate. The Fe and ZnO layers are chosen to satisfy the condition on refractive indexes $\frac{{{\boldsymbol{n}}}_{1}{{\boldsymbol{n}}}_{3}}{{{\boldsymbol{n}}}_{2}}=\sqrt{{{\boldsymbol{n}}}_{4}\cdot {{\boldsymbol{n}}}_{{\bf{a}}{\bf{i}}{\bf{r}}}}$ to achieve zero reflection surface for a 3 layered system^24^ (**b**) SEM and AFM micrograph of P-type Si wafer after etching with KOH solution using optimized etching process parameters. (**c**) SEM micrographs showing the detailed geometry of etched pillars with an aspect ratio of 1.3.

It is well established that the presence of multiscale micro-nano feature size and impedance matching is desirable to enhance light trapping and hence absorbing capacity of materials^7–11^. Calculations have shown that array of CNTs have refractive index of 1.1^14^ Thus, the key to design a near perfect broad band absorber is to combine the impedance matching characteristics of carbon nanotubes in an optically graded multilayered assembly having varying feature size to facilitate multiple scattering and hence absorption of incident beam within the assembly. Here, the choice of optical gradation is motivated by the simple fact that the incident beam will bend towards the vertical axis (Fig. 1a) on travelling from a lower index medium (*n*_1_) to a medium with higher index (*n*_2_) as per Snell’s law (*n*_1_sin*θ*_1_ = *n*_2_sin*θ*_2_). Moreover, the reverse optical gradient will partially block the reflected beam to escape from the substrate through total internal reflection (TIR). The choice of materials at a particular location in the multilayer assembly shown in Fig. 1a is motivated by the condition $\frac{{{\boldsymbol{n}}}_{1}{{\boldsymbol{n}}}_{3}}{{{\boldsymbol{n}}}_{2}}=\,\sqrt{{{\boldsymbol{n}}}_{4}\cdot {{\boldsymbol{n}}}_{{\bf{a}}{\bf{i}}{\bf{r}}}}$ to create a zero reflection surface as well as to ensure impedance matching at the top layer.

## Results and Discussion

Consistent with the proposed design approach, chemical etching of polished P-type Si wafer using KOH (at 80 °C for 10 mins) is done to create nano-sized texture in the form of conical pillars as reported elsewhere^20^. After etching, the wafer is rinsed with DI water and blow dried. AFM and SEM micrographs in Fig. 1b show that the samples etched with KOH has uniform, large density conical pillars having aspect ratio 1.3 and angle between the sidewalls and base is approximately 55° (as shown in Fig. 1c).

**Figure 2:**
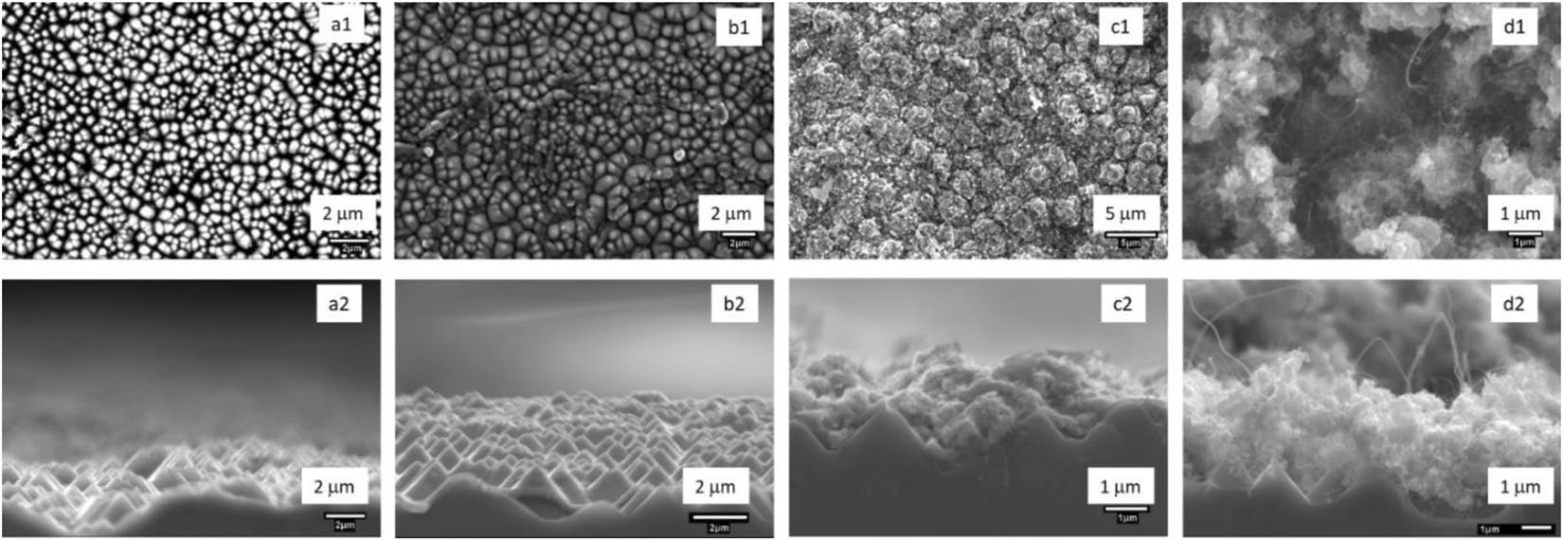
Surface morphology at different steps of fabrication of optically graded multilayered assembly Top row: SEM images of textured Si (a1), Iron coated (b1), Zinc coated (c1) and CNTs layer (d1) on P-type Si wafer viewed from the top. Bottom row: SEM images of textured silicon (a2), Iron (b2), Zinc (c2) coated and CNTs layer (d2) on P-type Si wafer viewed from the side.

Figure 2 compares the microstructure (top view in first row and side view in second row) of assembly after each fabrication step. The geometry of disordered array of etched conical pillars in SEM micrograph (Fig. 2a1,a2) found to be suitable to trap the light^9^. The optical index of etched Si wafer measured to be ~3.86 (see Table S3 in SI). The presence of any impurity after etching process could be detected from XRD analysis of etched wafer (Fig. S1 in SI). Next, layers of different materials are coated on top of the etched Si wafer to create a structure having optical as well as feature size gradation across the thickness of the multilayered assembly.

A thin layer of Fe (thickness = 400 nm) having optical index (*n* = 3.31, see Table S3 in SI) lower than etched Si is deposited on conical pillars using thermal vapor deposition. Micrographs in Fig. 2b1,b2 show that Fe layer takes the shape of the conical pillars and forms a wavy surface rather than a flat top surface. The corresponding XRD pattern in Fig. S1 expectedly shows the presence of characteristic peaks for Si and Fe. Since the optical index of ZnO (*n* = 1.94) measured to be lower than Fe (*n* = 3.31), a 400 nm thin layer of ZnO globules (see Fig. 2c1,c2) is formed on top of the Fe layer using hydrothermal process. The ZnO globules sits in the valley created between the Si pillars (Fig. 2c2) and adds another size scale in the assembly. To satisfy the zero reflection condition and impedance matching, a layer of CNTs is grown next on top of the ZnO layer inside thermal CVD reactor^14^. The thickness of CNT layer found to be 6 µm (Fig. 2d1–d3) with optical index of *n* = 1.17 and surface corrugations of 400–700 nm (see Fig. S2). The CNTs forms a noodle kind of structure with an average CNT diameter of 50 nm (Fig. 2d1) and offer a different feature size in multilayered assembly. Total thickness of optically graded multilayered assembly found to be about 6.8 µm (excluding wafer thickness) which is lower compared to many other reported optical absorbers^7,8,10,14^. The deposition of ZnO and CNT is confirmed from the characteristics peaks present in their respective XRD plots (Fig. S2c,d). Many absorbers are also been reported which are nonmetric/sub-micron thickness but on same time their absorption capacity has also been reduce drastically.

**Figure 3:**
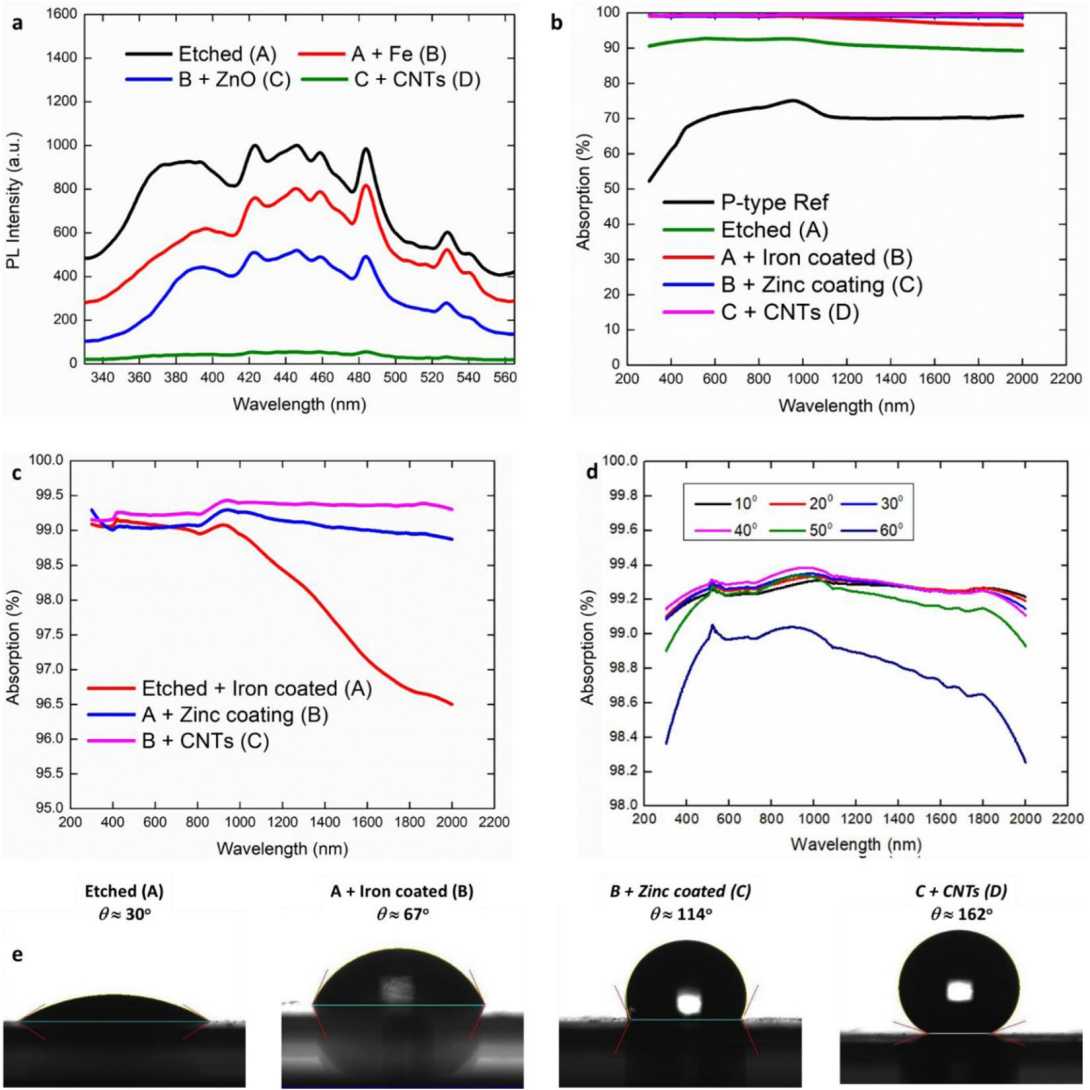
Photoluminescence and absorption spectrum followed by hydrophobicity study of optically graded assembly. (**a**) Photoluminescence spectra after deposition of each layers on the etched Si substrate. (**b**) UV-Vis-NIR spectra of P-type wafer after each processing step. (**c**) A zoom in view of (**b**). (**d**) Variation of surface absorption capacity as a function of incidence beam angle *ϕ* on optically graded multilayered surface. (**e**) Contact angle (*θ*) of water drops on substrate after each fabrication step.

To check the applicability of present design approach, photoluminescence (PL) properties of fabricated multilayer assembly are recorded (Fig. 3a) after deposition of each layer with excitation at 310 nm. The PL intensity is found to be maximum for textured silicon surface and keeps decreasing on addition of subsequent layers. PL data recorded after coating of top CNTs layer suggests that the multilayered system is indeed able to efficiently absorb the incident light in the given wavelength range. Encouraged by the results of PL measurements, the effect of optical gradation on the absorbing peformance of multilayered assembly in broader wavelength range of 300–2000 nm is investigated through UV-VIS-NIR spectroscopy analysis for un-polarized light. The variation of absorbing capacity as a function of processing steps carried out on P-type Si wafer is presented in Fig. 3b. The introduction of conical pillars increases the absorption up to 90% for etched wafer. The addition of other feature sizes after coating of Fe and ZnO improves the absorption of incident light to ~99% as compared in zoomed view of Fig. 3b presented in Fig. 3c. It is important to note that the absorption capacity of Fe layer coated etched wafer starts decreasing from 99% after visible range and reduces to 96% at 2000 nm. The introduction of ZnO globules makes the absorption stable at around 99% with a slight increase in 1000–1400 nm range. Coating of CNT layer on top of ZnO layer further increases the absorption of incident beam beyond 99% with higher absorption of 99.4% in NIR wavelength range. Thus, the optically graded surfaces behave like super absorber with around 99.4% absorption over the whole UV-VIS-NIR range (Fig. 3c) and not only in visible range. It can be inferred from the data compared in Fig. 3c that ZnO and CNT layers are more effective in improving the absorbance of graded assembly in 1000–2000 nm wavelength range in comparison to Fe layer. To get further insight, the absorption capacity of individual layers used in graded assembly is also studied and compared in Fig. S6. It is observed form the absorption spectrum (Fig. S6 in SI) that the deposition of either Fe or ZnO layer alone on the etched Si wafer is more effective in absorbing incident light as compared to deposition of same layers on plane polished wafer. The improved absorbing behavior with etched Si wafer may be attributed to multiple scattering by etched pillars. In addition, with pillars having a base angle of 55° leads to total internal reflection^21^ which helps in better light trapping of incident beam. It is observed in Fig. S6 that even though Fe and ZnO layers shows average absorption of more than 85% in visible range, a sudden decrease in absorption capacity is observed in case of Fe layer as compared to ZnO layer in infrared region. Which suggests that Fe is a good candidate for visible spectrum absorption and ZnO is better for light absorption in infrared range. It is interesting to note that the coating of CNT layer on etched Si wafer shows an almost constant absorbing capacity of 98% in the whole range. It simply means that the sudden drop observed in case of Fe and ZnO layer in NIR wavelength range can be countered by depositing a CNT layer on top of these layers. A slightly improved absorption behavior of about 99% in visible region is obtained by coating both Fe and ZnO layer in comparison to CNT coating on etched Si wafer. An assembly fabricated using all three layers provide a remarkable absorbing capacity of 99.4% in whole UV-Vis-NIR wavelength range due to the presence of multiscale feature sizes, multiple scattering, beam bending and total internal reflection (TIR) as discussed below.

Another key feature of the optical gradation is noticed by varying the angle of incidence of beam in UV-Vis-NIR measurements. Figure 3d shows that the absorption remains 99% higher for beam angle up to 50° and decreases slightly to 98.8–98.6% for 60° incidence angle. These observations show the potential of optical gradation approach with CNT as top layer to process super black absorbers for next generation solar cells in harnessing energy in 1000–1400 nm wavelength range.

The super black nature of multilayered graded surface can be attributed to a number of factors such as beam bending, total internal reflection and presence of multiscale feature sizes etc. With incident light traveling from rare to denser medium in optically graded assembly, it will slow down and bend towards normal due to higher molecular interaction in denser medium and Snell’s law. The bend angle *θ*_b_ (*θ*_b_ = *θ*_i_ − *θ*_r_) of incident beam while passing through different layers has been compared in Table ST2 for two incident angle *θ*_i_ of 10° and 60°. A maximum inward bending *θ*_b_ of 3° and 21° for *θ*_i_ of 10° to 60° is obtained when the light travels from CNT to ZnO layer due to largest difference in optical index of these two consecutive layers. Table ST3 tabulates the critical angle *θ*_c_ for reflected beam at each interface when it is travelling from Si wafer towards Air. Lower *θ*_c_ in Table S4 for Fe/ZnO and ZnO/CNTs layer interface reveals that most of the reflected light will be trapped inside assembly due TIR of reflected beam having angle more or equal to 35° to 37° respectively.

Increased optical path length of photon due to scattering of incident light from nano structured conical pillars on etched wafer also aids in light trapping in multilayered assembly^12^. In addition, the existence of multiscale feature sizes due Fe, ZnO and CNT layers triggers multiple reflections and scattering within the textured area^7,8,11^. As a consequence, both longer as well shorter wavelengths of incident beam gets absorbed by the optically graded surfaces. The combined effect of all the interactions of incident beam with the multilayered optically graded assembly having multiple feature size results in a near perfect black body over a wide range of wavelength and incident angle. It is to be noted here that the absorption capacity of ~99.4% achieved in this work is primarily limited by the presence of air/CNT top layer and other interfaces in the multilayer assembly. These interfaces exist due to the discretely varying optical indexes of individual layers. A continuous optical gradation across the thickness may help us to further improve the absorption capacity beyond 99.4%.

Environmental assisted corrosion due to moisture absorption is another key concern for the degradation of absorbing surfaces^22^. To this end, wetting behavior of multilayered assembly is investigated by measuring contact angle (CA) of water drops using goniometer in Fig. 3e. CA values recorded after each fabrication step reveal the superhydrophobic (Contact angle *θ *≈ 162°) behavior of fabricated assembly. Moreover, a roll off angle (the angle at which water drops start rolling without sticking on the surface) of 5° indicates that the processed surface is not only superhydrophobic, it is also of self-cleaning with water drops forming Cassie-Baxter type configuration^23^. This is a very promising aspect regarding industrially viable applications of processed super absorbers as it will avoid the degradation of coatings over time by moisture absorption from surrounding environment. The near perfect optical absorption and self-cleaning behaviour of multilayer surface show the applicability and potential of present design approach. It is envisaged that it will be a significant step forward in designing near-perfect absorbers for an even broader wavelength applications.

In summary, we have shown that it is possible to fabricate near perfect broadband absorber on etched Si wafer through well designed optical gradation scheme. As a proof of concept, optically graded absorber shows more than 99% absorption in the 300–2000 nm wavelength region with beam incident angle varying from 10–60°. The inward bending of beam due to increasing optical index in the direction of beam, impedance matching by top CNT layer and multiple scattering from multiscale feature size present across the thickness are primarily responsible for the observed optical behavior of multilayered assembly. Another advantage of the present design approach is that the assembly shows superhydrophobic self-cleaning behavior making it quite attractive for next generation solar energy and antireflection coatings applications. As the fabrication steps are easier and cost effective in comparison to other reported fabrication techniques^4,7^, the large scale fabrication of proposed absorber is possible with just increase in the chamber size of thermal evaporator.

## Experimental Section

### Substrate fabrication

P type silicon wafers 2 × 2 cm having orientation 100 and resistivity 1–10 ohm-cm are used for present work. All wafers are cleaned by 20 min sonication in IP followed by DI water. Wafers are dipped in solution with concentration 1:10 HF and DI water for 30 sec to remove native oxide layer, followed by sonication in IP for 15 min and are dried by hot air gun. KOH solution is prepared by adding 5 g KOH in 20 ml DI water at 80 °C. KOH solution is stirred at 400 rpm for growth of conically shaped pillars on polished silicon surface. Silicon wafers are dipped in the solution, with wafer polished side kept perpendicular to the flow direction for maximum etching to take place in vertical direction. Etching time is varied from 1 min to 10 min. After etching the samples are rinsed with DI water and blow dried. After etching layer of 400 nm Fe layer followed by 98% pure zinc is deposited by physical vapour deposition (PVD). Subsequently, ZnO nanostructures are grown by hydrothermal method at 60 °C for 6 hours. Fe layer of 25 microns is coated as catalyst for CNT. CNTs are grown by thermal CVD process using argon, hydrogen and acetylene as precursor gases at 820 °C.

### Optical measurements

Absorption spectra are taken (PerkinElmer LAMBDA 950 UV/Vis/NIR spectrophotometer, USA) in both transmission and reflection mode. Ellipsometric measurements are performed using Woollam Spectroscopic Ellipsometer (M 2000-F, USA). Surface morphology is observed by scanning electron microscopy (Jeol 6610LV, Japan). Atomic force microscopy (Bruker, USA) is performed on silicon sample for verification of surface texture. XRD data is obtained from (Panalytical X, peart Pro, MPD system, United Kingdom) with an X-ray source of Cu (1.54 Å). Contact angle study is performed by Contact Angle Analyzer (First Ten Angstroms, USA) and photoluminescence with excitation at 310 nm by (Bruker, USA) PL spectrometer.

## Electronic supplementary material

Supporting Information
