# Supplementary material for: Optically Graded Ultra Dark Absorber for Visible and Near-infrared Wavelength Range
Source: Sci Rep. 2018 Aug 17;8:12312. doi: 10.1038/s41598-018-30844-5 (PMC6098028; doi:10.1038/s41598-018-30844-5)
Supplement: Supplementary file 1 — Supporting Information [file 41598_2018_30844_MOESM1_ESM.pdf]

Supporting Information

Optically Graded Ultra Dark Absorber for Visible and Near-infrared Wavelength Range

Prabhat K. Agnihotri*[‡] *, Viney Ghai*[‡] *and Harpreet Singh*

(‡ Equal Contributors)

Department of Mechanical Engineering
Indian Institute of Technology Ropar
Nangal Road, Rupnagar-140001, Punjab, India

*Correspondence and requests for materials should be addressed to Prabhat K. Agnihotri and Harpreet Singh (email: prabhat@iitrpr.ac.in, harpreetsingh@iitrpr.ac.in)

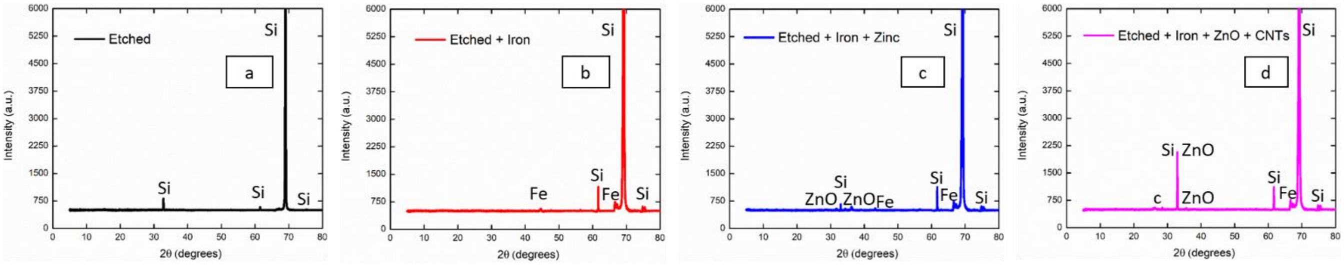

Figure S1: XRD pattern of graded assembly taken after coating of each layer. XRD pattern of **(a)** Etched Si wafer, **(b)** Fe coated etched Si wafer, **(c)** After ZnO coating on top of Fe layer and **(d)** After deposition of CNTs on top of ZnO layer.

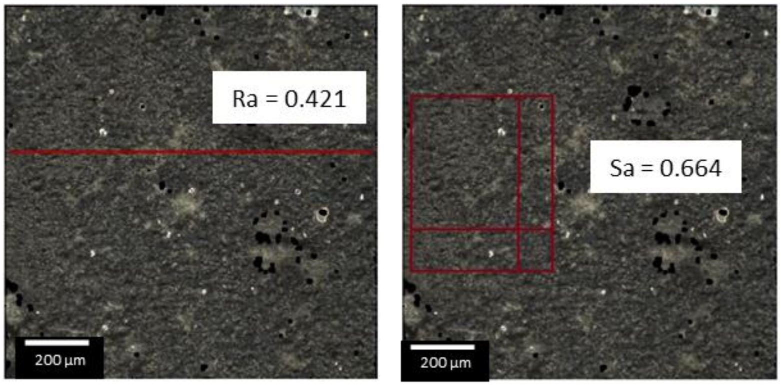

Figure S2: Surface corrugations of CNT layer.

Table S3: Measured real (n) and imaginary (k) part of complex refractive index of different layers used to fabricate optically graded multilayer assembly.

**S No.**	**Substrate**	***n***	***k***
1	Si	3.9632	0.02269
2	Si etched	3.8697	0.02545
3	Fe	3.31	0.02826
4	ZnO	1.9423	0.02175
5	CNT	1.17	0.02652

Table S4: Calculated beam bend angle ($\theta_b = \theta_i - \theta_r$) as per Snell's law ($n_1 \sin\theta_1 = n_2 \sin\theta_2$) of incident beam on travelling through different layers of optically graded assembly. Two beam incident angles of 10° and 60° are used in this calculation.

Beam Bending Study			
Gradation	Incident Angle (θ_i)	Refracted Angle (θ_r)	Bending Angle $\theta_b = (\theta_i - \theta_r)$
Air to CNT (θ_1)	60°	47.76°	12.24°
CNT to ZnO (θ_2)	47.76°	26.34°	21.42°
ZnO to Fe (θ_3)	26.34°	15.09°	11.25°
Fe to Etched Si (θ_4)	15.09°	12.86°	2.23°
Gradation	Incident Angle (θ_i)	Refracted Angle (θ_r)	Bending Angle $\theta_b = (\theta_i - \theta_r)$
Air to CNT (θ_1)	10°	8.5°	1.5°
CNT to ZnO (θ_2)	8.5°	5.1°	3.4°
ZnO to Fe (θ_3)	5.1°	2.99°	2.11°
Fe to Etched Si (θ_4)	2.99°	2.56°	0.43°

Table S5: Critical angle ($\boldsymbol{\theta_c = \sin^{-1}\left(\frac{n_r}{n_i}\right)}$) for total internal reflection of reflected beam on passing through different layers in optically graded assembly.

Critical Angle study	
CNT to Air (θ_{CA})	58.75°
ZnO to CNT (θ_{ZC})	37.04°
Fe to ZnO (θ_{FZ})	35.93°
Etched to Fe (θ_{EZ})	58.79°

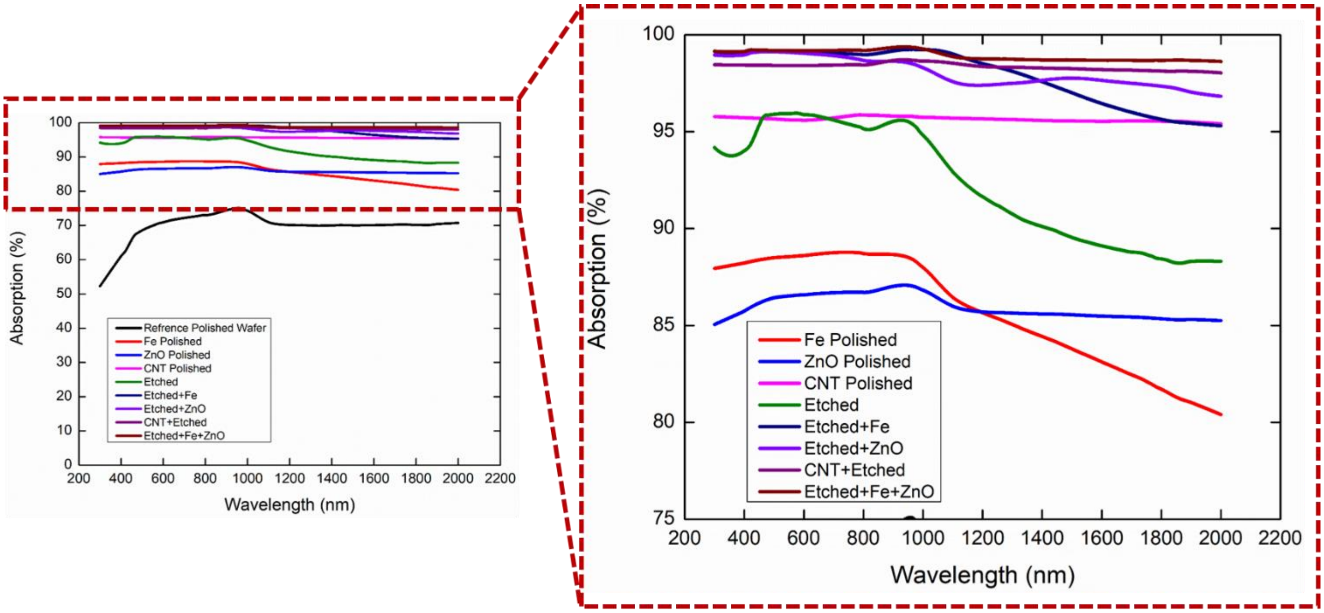

Figure S6: Absorption spectrum of Fe, ZnO, CNTs on polished and etched Si wafer to study individual absorption behavior.
